# Effect of a Recombinant Human Basic Fibroblast Growth Factor 2 (rhFGF-2)-Impregnated Atelocollagen Sponge on Vertical Guided Bone Regeneration in a Rat Calvarial Model

**DOI:** 10.3390/dj13040177

**Published:** 2025-04-20

**Authors:** Keisuke Kogure, Akira Hasuike, Risa Kurachi, Yasuyuki Igarashi, Masataka Idesawa, Shuichi Sato

**Affiliations:** 1 Division of Applied Oral Sciences, Nihon University Graduate School of Dentistry, Tokyo 101-0062, Japan; 2 Department of Periodontology, Nihon University School of Dentistry, Tokyo 101-8310, Japan; 3 Dental Research Center, Nihon University School of Dentistry, Tokyo 101-8310, Japan

**Keywords:** alveolar ridge augmentation, bone regeneration, dental implants, skull, X-ray microtomography

## Abstract

**Background/Objectives**: Achieving a sufficient volume of augmented bone, particularly for vertical bone regeneration, remains challenging. This study investigated the use of basic fibroblast growth factor 2 (FGF-2) to promote bone augmentation beyond the skeletal envelope in the rat calvarium. **Methods**: Seven rats were included in the study, with bilateral experimental sites in the calvarium. Two plastic caps were placed in the calvarium, containing either 0.3% FGF-2 with an atelocollagen sponge or an atelocollagen sponge alone as a control. Bone augmentation within the plastic caps was evaluated using micro-computed tomography (micro-CT) scans and histological sections. Micro-CT measurements, including bone volume measurements, were obtained at 1 week to 12 weeks after surgery. At 12 weeks, the area and height of the newly formed bone were evaluated using histological sections. **Results**: Starting at 8 weeks after surgery, the volume of the newly formed bone in the 0.3% FGF-2 group was significantly greater than that in the control group. At 12 weeks, histomorphometric analyses revealed that the area and height of the newly augmented bone were 35.6% and 41.9%, respectively, in the FGF-2 group, compared with 9.1% and 13.4%, respectively, in the control group. **Conclusions**: The inclusion of 0.3% FGF-2 in atelocollagen sponge enhanced vertical bone augmentation beyond the skeletal envelope in the rat calvarium. These findings have potential applications for improving bone regeneration outcomes in dental implant procedures.

## 1. Introduction

Dental implants have become increasingly popular owing to their superior effectiveness over prostheses supported by natural teeth or adjacent oral soft tissues in edentulous areas. However, the long-term success of these implants depends on sufficient bone volume (BV) and bone quality to provide structural support and stability [1]. In cases involving an inadequate alveolar ridge, such as jaw atrophy, periodontal disease, traumatic bone loss, or post-tumor resection, additional interventions are needed to augment both hard and soft tissues before or simultaneously with dental implant placement [2,3]. Among these, guided bone regeneration (GBR) has been extensively investigated and is widely regarded as the most frequently used intervention for both horizontal and vertical ridge augmentation [4,5,6,7]. Although autografts remain the gold standard for GBR, they present limitations, including donor-site morbidity, limited availability, and potential complications. Allografts offer an alternative, but they carry risks of immune rejection and disease transmission. Despite their availability and reproducibility, emerging biomaterials often fail to fully integrate into the host bone and may develop inhibitory fibrous encapsulation [8]. Achieving a sufficient volume of augmented bone using GBR for implant placement remains a challenge, particularly for vertical bone regeneration. Additionally, uncertainty persists regarding the functionality of GBR-generated bone compared with that of native autogenous bone.

To address these challenges, various bone-inducing growth factors have been evaluated; these can potentially expedite implant placement and improve predictability [9,10]. Osteoinduction, in combination with osteoconductive bone grafts, enhances bone regeneration and tissue healing. Basic fibroblast growth factor 2 (FGF-2) is a particularly promising candidate for tissue regeneration [11]. FGF-2 is a member of the fibroblast growth factor family and consists of polypeptides that play crucial roles in regulating the proliferation and differentiation of various cell types. This growth factor has been extensively used to treat decubitus ulcers [12,13]. Initial research demonstrated that FGF-2 stimulates both the proliferation and migration of cells derived from the periodontal ligament [14,15], prompting investigations into recombinant human FGF-2 (rhFGF-2) as a potential therapeutic option for intrabony periodontal defects [16,17,18]. This resulted in the formal approval of 0.3% rhFGF-2 in 2016 for use in periodontal regenerative medicine in Japan [19].

Building on its success in periodontal regenerative therapy, the scope of research has expanded to include the broader potential of rhFGF-2 in bone healing and regeneration [20,21,22]. rhFGF-2 operates through multiple mechanisms to enhance bone formation: it maintains osteoblast precursors in a proliferative state while significantly promoting angiogenesis, which is crucial for nutrient and oxygen supply in the newly formed bone tissue. Additionally, rhFGF-2 stimulates the secretion of other growth factors, such as vascular endothelial growth factor and hepatocyte growth factor, further enhancing vascular development and creating a favorable microenvironment for bone regeneration. The ability of growth factors to promote cell proliferation and angiogenesis, which was first observed in periodontal applications, has proven to be equally valuable for general bone regeneration. When applied to bone defects, rhFGF-2 demonstrates remarkable effectiveness in enhancing bone formation through increased vascularization and osteogenic cell recruitment. Beneficial effects are observed in combination with osteoconductive bone minerals, creating a synergistic environment that promotes both the quantity and quality of regenerated bone [20,21,22].

Despite these encouraging findings, few studies have investigated the role of rhFGF-2 in vertical bone regeneration. However, some studies have evaluated its effectiveness in vertical augmentation, particularly in the absence of additional bone mineral particles. To address these limitations, preclinical studies using standardized animal models with rhFGF-2 are needed. Our research group developed a standardized vertical GBR experimental model using plastic caps placed on the rat calvarium [23]. This model is particularly suitable for studying vertical bone regeneration for several reasons: the calvarium provides a well-defined, flat cortical bone surface with minimal anatomical variations between specimens, allowing for standardized and reproducible defect creation; the limited vascularity and predominant cortical structure of the calvarium closely mimic the challenging conditions encountered in atrophic jaw bones; the model eliminates functional loading variables, enabling the isolated assessment of biological bone regeneration capacity; the plastic caps create a protected space that simulates the clinical GBR technique used in vertical ridge augmentation procedures. This model provides an important basis for translational research and medical technology development, enabling the evaluation of various substances, including the effects of bone substitutes [24], hormones [25], and smoking [26], on the volume of newly augmented bone.

The objective of this study was to investigate the effects of rhFGF-2 on vertical bone augmentation beyond the skeletal envelope using a rat calvarial GBR model. This study is expected to contribute to the application of rhFGF-2 in enhancing bone augmentation and improving dental implant placement outcomes.

## 2. Materials and Methods

### 2.1. Animals

Seven male Fischer rats (12 weeks old, weighing 200–250 g) were used in this study. The primary outcome measure was the percentage of BV. The required sample size was determined using GraphPad Prism version 10.0 (GraphPad, San Diego, CA, USA) with an alpha level of 0.05 and a statistical power of 90%. This calculation was based on data from a previous study [27], which reported newly formed bone percentages of 46.6 ± 7.8% and 27 ± 1% for occlusive and non-occlusive barriers, respectively. Using these values, an effect size (Cohen’s d) of 3.52 was obtained, indicating that at least six animals were necessary to detect statistically significant differences between the groups. Seven animals were included to compensate for potential experimental loss. The rats were housed in a controlled facility with environmental conditions maintained at 22 °C, 55% humidity, and a 12 h light/dark cycle. The rats had unrestricted access to food and water throughout the study period.

### 2.2. Preparation of FGF-2 Incorporated in the Atelocollagen Sponge

Freeze-dried rhFGF-2 (Kaken Pharmaceutical Co. Ltd., Tokyo, Japan) was thawed and diluted with saline. A non-mineralized absorbable atelocollagen sponge (Teruplug; Terumo, Tokyo, Japan) was used as a carrier for rhFGF-2. In tissue engineering, animal-derived collagen is mostly used and hence may cause immunogenic reactions in humans. Tissue engineering frequently utilizes animal-derived collagen, which poses a risk of immunogenic reactions in humans. Atelocollagen is a low-immunogenic collagen derivative in which the N- and C-terminal telopeptide components of collagen molecules have been enzymatically removed [28]. These telopeptides are the major antigenic determinants in collagen-based biomaterials, and their removal significantly reduces immunogenicity compared to standard collagen, making atelocollagen more biocompatible and less likely to trigger immune responses when implanted. The atelocollagen sponge used in this study is derived from young bovine dermal collagen and formed into a bullet-like shape, consisting of types I and III collagen [29]. The biomaterial uniquely combines two distinct forms: fibrillar atelocollagen (self-assembled into regularly aggregated fibrous structures under physiological conditions) and heat-denatured atelocollagen (produced through thermal denaturation in aqueous solution, disrupting its triple-helical structure) [29]. Fibrillar atelocollagen exhibits superior physical stability and biological durability when implanted in vivo. In contrast, heat-denatured atelocollagen demonstrates enhanced cell infiltration capabilities. This optimized scaffold containing both forms is expected to support cell migration, proliferation, and differentiation for both soft and hard tissue regeneration applications. Atelocollagen sponges were impregnated with 20 μL of 0.3% rhFGF-2 for the test group. The selection of 0.3% rhFGF-2 concentration in our study was based on periodontal clinical evidence from previous studies. Kitamura et al. conducted a multicenter, randomized, double-blind, and placebo-controlled clinical trial involving 253 patients with periodontitis [18]. Their dose-finding study evaluated three different concentrations of rhFGF-2 (0.2%, 0.3%, and 0.4%) for periodontal bone regeneration. The results demonstrated that all rhFGF-2 concentrations showed significant improvement over placebo, but the 0.3% concentration exhibited optimal efficacy and provided the highest percentage of bone fill. Dose–response analysis revealed that bone formation plateaued at this concentration, with no additional benefits observed at higher concentrations. This finding led to the formal approval of 0.3% rhFGF-2 as a periodontal regenerative medicine in Japan in 2016, as mentioned in the introduction. Based on the established optimal concentration for periodontal regeneration, we selected 0.3% rhFGF-2 for our vertical bone augmentation model. As a control, rhFGF-2-free atelocollagen sponges were prepared in a similar manner through immersion with 20 µL of sterile saline solution. The atelocollagen sponges were fitted to the plastic caps.

### 2.3. Surgical Procedures

The rats were pre-medicated with isoflurane anesthesia through inhalation and were then administered general anesthesia via the intraperitoneal injection of a mixture consisting of 0.15 mg/kg dexmedetomidine hydrochloride, 2.0 mg/kg midazolam, and 2.5 mg/kg butorphanol tartrate. To control bleeding and provide additional anesthesia, a 0.5-mL intraperitoneal injection of lidocaine (Xylocaine; Astra Zeneca, Osaka, Japan) diluted at a ratio of 1:80,000 was administered.

**Figure 1:**
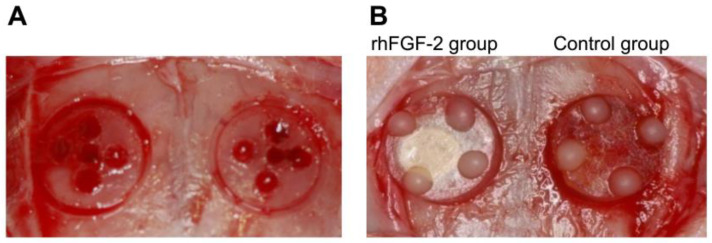
(**A**) A circular groove (5 mm) was made using a trephine burr, and five small holes were drilled using a round burr. (**B**) A plastic cap was fixed to each groove. Left: rhFGF-2 group. Right: Control group. rhFGF-2, recombinant human fibroblast growth factor 2.

After shaving and disinfecting the region between the eyes and the posterior end of the skull with 70% ethanol swabs, a midline incision of 6.0 cm was made, and a mucoperiosteal flap was elevated with a small sharp periosteal elevator to expose the cranial vertex. A circular groove with an inner diameter of 5 mm was created on each side of the midsagittal suture using a trephine burr, with constant irrigation with sterile saline. The midsagittal suture was not included in the bone defects to avoid its contribution to bone healing and to limit the risk of damage to the superior sagittal sinus. Five small holes were drilled using a round burr to induce bleeding (Figure 1A). A cylindrical plastic cap measuring 1.5 mm in height and 4.4 mm in diameter was placed on both sides of the circular grooves, and composite resin landmarks were attached to the top of the plastic caps. One of the two bilateral circular grooves was selected as the test site for setting up a plastic cap with 0.3% rhFGF-2-incorporated atelocollagen sponges. The other side, used as the control, was treated with saline-immersed atelocollagen sponges. After exhaustively washing the surgical area with sterile saline to remove any bone scraps, plastic caps with rhFGF-2 or saline were pressed into the circular grooves (Figure 1B). After surgery, the skin and periosteum were repositioned and sutured using simple resorbable interrupted sutures (VSORB 4-0; Washiesu Medical, Tokyo, Japan). The day of surgery was designated as day 0. The rats were euthanized at 12 weeks postoperatively.

### 2.4. Micro-Computed Tomography Imaging

**Figure 2:**
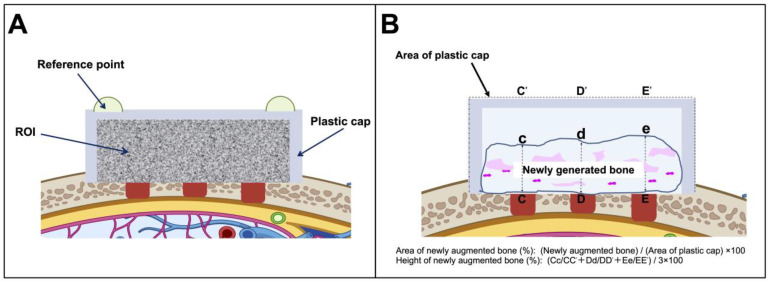
Schematic images of the animal model. (**A**) Schematic of the plastic cap and area visualized using micro-computed tomography and the measurements obtained. (**B**) Measurements of histological sections. ROI, region of interest.

To evaluate mineral volume at the surgical sites, an in vivo micro-computed tomography (micro-CT) scanner (R_mCT; Rigaku, Tokyo, Japan) was used, allowing imaging without requiring the rats to be euthanized. A base plate was connected to the X-ray source using an image intensifier, whereas the I-arm was driven by a direct-drive motor and rotated within the vertical plane. The imaging parameters were configured as follows: pixel resolution, 480 × 480; voxel dimensions, 30 × 30 × 30 µm; slice thickness, 120 µm; tube voltage, 90 kV; tube current, 100 µA; exposure duration, 17 s. Images were reconstructed using custom i-View software (Kitasenjyu Radist Dental Clinic; i-View Image Center, Tokyo, Japan). BV within the plastic cap was quantified from the voxel data using proprietary analysis software. The software calculated the gray values and voxel count corresponding to those values within the defined regions of interest (ROIs) (Figure 2A). On day 0, the ROI was set to include the entire region within the plastic caps, based on the reference points fabricated from the resin on the plastic caps. Subsequent imaging was performed every 2 weeks, setting ROIs as identical as possible to those of day 0 using the reference points and anatomical features. A histogram was generated, with the x-axis indicating the X-ray absorption rates and the y-axis representing the CT voxel counts. Peaks corresponding to hard and soft tissues were identified, and the threshold was established at the trough between these peaks. Voxels with absorption rates above this threshold were counted. The BV was then derived by multiplying the number of such voxels by the individual voxel volume. Measurements were conducted in each ROI on the first postoperative day and, subsequently, on a weekly basis under identical scanning conditions. The increase in BV was determined by subtracting the initial day 1 value from each subsequent measurement. This increase was interpreted as an indication of new bone formation.

### 2.5. Histological and Histomorphometric Analyses

The rats were euthanized by CO_2_ gas inhalation at 8 and 12 weeks after the final micro-CT scan. The calvarial bone containing the bone defects or the fixed plastic cap was excised, fixed in 10% neutral-buffered formalin, dehydrated, embedded in paraffin wax, and sectioned into 5 µm slices for hematoxylin and eosin staining. One sagittal decalcified ground section from the center of the plastic cap was prepared using a microtome. The sections were assessed histologically and morphometrically using a light microscope equipped with a morphometric system (Model BFH-142; Olympus, Tokyo, Japan) connected to a personal computer. Histomorphometric data from the central section of each specimen were recorded using a computerized image analysis system. Images were taken at 40× magnification, digitized, and processed using a charge-coupled device linear photodiode array interfaced with a computer. Measurements were extracted from the digital images using interactive image-processing software.

For each central histological section, we calculated the percentage of newly generated bone area relative to the area bound by the plastic cap and parent bone, with the latter designated as 100%. We determined the cross-sectional area of the newly generated bone, which was expressed as a percentage of the height and total area of a representative histological section (Figure 2B). These sections were compared with the corresponding micro-CT images to ensure the alignment of specific homologous anatomical features in the bony anatomy. All histological sections were evaluated by a single examiner blinded to the study conditions.

### 2.6. Statistical Analyses

The means and standard deviations were calculated for the BV, defect closure rate, percentage of newly formed bone area, and bone height. The Wilcoxon signed-rank test was used to compare the parameters between the rhFGF-2 and control groups. Statistical significance was set at *p* < 0.05. GraphPad Prism version 10.0 was used for all the statistical analyses.

## 3. Results

Healing progressed uneventfully without complications in all animals. No signs of postoperative infection were observed in any of the animals used in the experiment.

### 3.1. Micro-CT Images

**Figure 3:**
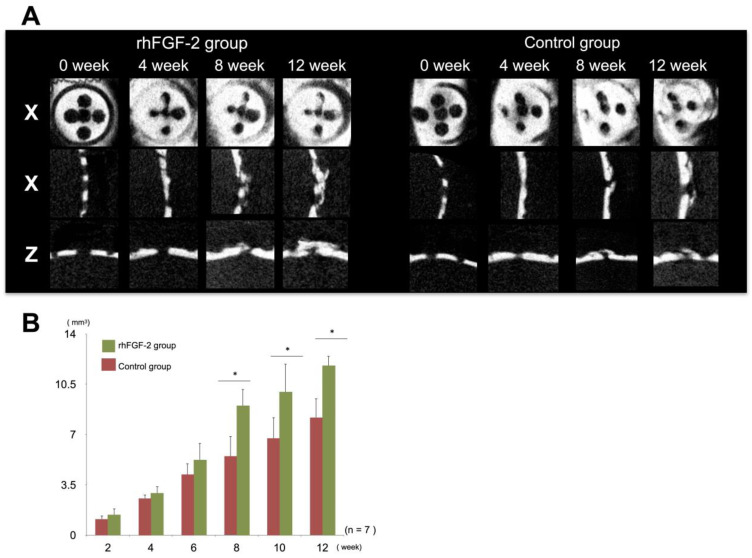
Micro-computed tomography (micro-CT) analysis. (**A**) Representative micro-CT images of a plastic cap in the rhFGF-2 and control groups. X, horizontal; Y, sagittal; Z, coronal. (**B**) Bone volumes in the rhFGF-2 and control groups. * *p* < 0.05. rhFGF-2, recombinant human fibroblast growth factor 2.

Analyses of the micro-CT images indicated that the radiopaque contrast gradually increased in both the experimental and control groups. The radiopaque layer in the rhFGF-2 group was slightly thicker than that in the control group at 8 and 12 weeks (Figure 3A). Morphometric analyses using micro-CT images showed that BV increased in a time-dependent manner in both the rhFGF-2 and control groups. In the rhFGF-2 group, the radiopaque area increased rapidly from week 8, and there were statistically significant differences between the groups at weeks 8, 10, and 12 (*p* < 0.05). At 12 weeks, approximately half of the ROIs inside the caps in the rhFGF-2 group were filled with radiopaque areas (Figure 3B).

### 3.2. Histological Observation and Histomorphometric Analysis

**Figure 4:**
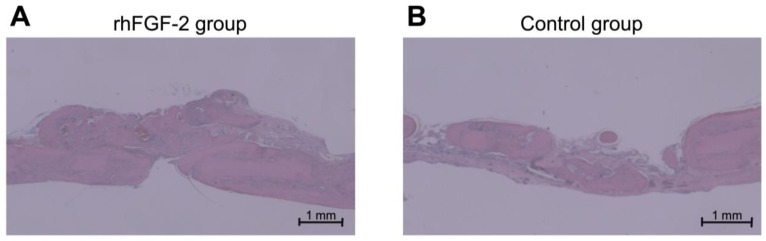
Representative histological sections at week 12. (**A**) rhFGF-2 group (original magnification ×40, scale bar: 1 mm). (**B**) Control group (original magnification ×40, scale bar: 1 mm). rhFGF-2, recombinant human fibroblast growth factor 2.

Histological sections obtained at 12 weeks showed rigorous bone formation in the rhFGF-2 group (Figure 4A). Although complete osseous regeneration was not evident in the control cohort, bone penetration sealed with newly formed bone, with the trabecular architecture encapsulated by cortical bone formation, both at the external periosteal surface and at the endocranial surface directly superior to the dura mater [30]. Histomorphometric analyses revealed that in the rhFGF-2 group, new bone occupied more than one-third of the area within the cap, with statistically significant differences between the groups (*p* < 0.05; Table 1).

## 4. Discussion

This study demonstrated that the incorporation of 0.3% rhFGF-2 into an atelocollagen sponge significantly enhanced vertical bone augmentation beyond the skeletal envelope in a rat calvarial model. Both the BV and vertical height of the newly formed bone were greater in the rhFGF-2 group than in the control group, with significant differences emerging after 8 weeks postoperatively, as confirmed by micro-CT. The successful vertical bone growth achieved in this study, which reached approximately two-thirds of the plastic cap height in the rhFGF-2 group compared with one-third of that in the control group, represconfirmedents a significant advancement in addressing one of the most challenging aspects of bone regeneration in dental implantology. This is particularly noteworthy because vertical augmentation has not traditionally shown highly predictable outcomes.

A significant finding was the time-dependent nature of bone formation, with the differences becoming statistically significant after 8 weeks. Only one previous animal study has investigated the efficacy of rhFGF-2-loaded atelocollagen sponge composite scaffolds for vertical bone regeneration, comparing low-dose (3 µg) versus high-dose (15 µg) rhFGF-2 applications. The investigators reported that while specimens in the high-dose cohort exhibited accelerated osteogenesis during the immediate postoperative period (1 week to 2 weeks), the low-dose group demonstrated time-dependent bone regeneration, which parallels our observations. Given that our dosage was closer to the low-dose parameters established in the aforementioned study, our findings corroborate and extend their conclusions regarding the temporal dynamics of rhFGF-2-mediated osteogenesis. Although it is difficult to directly apply the results of small animal studies to human clinical practice, the observed temporal dynamics are clinically relevant for treatment planning and suggest that shorter healing periods might be insufficient for optimal bone regeneration, even with growth factor enhancement. The continued increase in BV for up to 12 weeks indicated that the biological effects of rhFGF-2 persisted beyond the initial healing phase.

One notable aspect of this study was the use of rhFGF-2 without additional bone mineral particles, demonstrating that significant vertical augmentation can be achieved through growth factor stimulation alone when combined with appropriate space maintenance. We used atelocollagen sponges as scaffolds with rhFGF-2 for bone regeneration. The applications of various collagen-based carriers containing FGF-2 in GBR have been extensively discussed. We previously applied the same absorbable atelocollagen with 0.3% rhFGF-2 for critical [31] and noncritical [32] bone defects in rat calvaria. In these previous studies, rhFGF-2-impregnated atelocollagen sponges increased blood vessel and bone formation in critical- and noncritical-sized defects. Kobayashi et al. reported that a composite scaffold consisting of an rhFGF-2-loaded collagen gel sponge promotes bone augmentation in cranial bone defects [30]. Histological observations indicated that the rhFGF-2-loaded collagen scaffolds rapidly enhance the proliferation of osteoblasts and fibroblasts within the collagen matrix. Another study demonstrated that collagen-binding rhFGF-2 combined with a composite material effectively facilitates bone regeneration in horizontal bone defects in rats, likely because of the sustained release of rhFGF-2 [33].

Although our rhFGF-2 approach without bone mineral particles shows promise, its clinical translation requires addressing both biological and mechanical considerations. In our study, we used a plastic cap to surround the atelocollagen sponge, which provided crucial space maintenance and structural stability during the experimental period, a strategy that may not be directly applicable in clinical settings. Bone healing involves complex interactions among osteoblasts, osteoclasts, mesenchymal stem cells, and immune cells during the critical early inflammatory phase. Our rhFGF-2-loaded atelocollagen sponges likely modulate hematoma formation and immune cell recruitment but may face mechanical stability challenges in clinical settings. Collagen membranes deserve greater attention in this context because their specific characteristics (porosity, surface topography, chemical composition, and stiffness) directly influence soft tissue exclusion and space maintenance for regeneration [34]. Future studies should investigate the synergistic cooperation between rhFGF-2-loaded collagen sponges and optimized membranes, where membranes can replace our experimental plastic cap by providing structural stability while preventing fibrous tissue encapsulation and preventing sponges from delivering biological stimulation. The carrier delivery system and rhFGF-2 administration are critical factors for optimizing therapeutic outcomes. Fukuba et al. demonstrated that acidic gelatin sponges with β-tricalcium phosphate (β-TCP) and rhFGF-2 result in greater new bone formation in ridge augmentation than that observed using basic gelatin sponges [20]. This outcome was attributed to the superior controlled release properties of the acidic gelatin carriers. Their findings were further corroborated in a 2021 study that investigated ridge preservation in extraction sockets with dehiscence defects, where the combination of β-TCP and rhFGF-2 better preserved the ridge dimensions and reduced bone resorption compared with β-TCP alone [22]. Histological analysis indicated that the regions treated with rhFGF-2 exhibited higher proportions of mineralized bone and more appropriate bone architecture, suggesting enhanced tissue regeneration. Additionally, other alternative carriers, including hydrogels and synthetic polymers, warrant the evaluation of their effects on early-stage healing and integration with the host bone, potentially addressing the current limitations in which biomaterials fail to fully integrate and become surrounded by fibrous tissue. Additional research is necessary to confirm whether absorbable collagen is indeed superior and to clarify the precise requirements for space maintenance, which would allow us to fully optimize rhFGF-2′s regenerative potential. These findings may have significant implications for enhancing the efficacy of rhFGF, streamlining current GBR protocols, and potentially reducing the need for bone-substitute materials.

This study has several limitations. First, although standardized and well-established, the rat calvarial model may not fully replicate the clinical conditions of the human oral environment, particularly in terms of mechanical loading and blood supply. Second, the study duration of 12 weeks, which is sufficient to demonstrate significant bone formation, may not have captured the complete remodeling process. Third, although statistical significance suggests robust treatment effects, the sample size was relatively small. Finally, although enhanced bone formation was observed in the rhFGF-2 group, the biological mechanisms underlying the vertical bone augmentation were not directly investigated. The enhanced bone formation observed in the rhFGF-2 group could be attributed to several biological mechanisms. FGF-2 stimulates angiogenesis and promotes the proliferation and migration of osteogenic cells, which are crucial for bone regeneration [35]. The gradual increase in BV over time, as evidenced by both micro-CT and histological analyses, suggests that FGF-2 maintains its biological activity throughout the healing period when delivered via an atelocollagen sponge. This sustained effect is particularly important for vertical bone augmentation, in which space maintenance and continuous osteogenic stimulation are essential.

## 5. Conclusions

Our study demonstrates the significant potential of 0.3% rhFGF-2 for vertical bone augmentation. Using a standardized rat calvarial model, we found that rhFGF-2 delivered through an atelocollagen sponge carrier achieved substantial vertical bone regeneration, with histomorphometric analyses revealing a 35.6% new bone area in the rhFGF-2 group compared with 9.1% in the controls, and vertical bone height reaching 41.9% versus 13.4%, a more than three-fold increase in vertical bone formation. These findings suggest that, with proper space maintenance, rhFGF-2 can effectively promote vertical bone regeneration without requiring additional bone mineral particles. The time-dependent nature of bone formation, with significant differences emerging after 8 weeks (*p* < 0.05), provides valuable insights for clinical treatment planning. In addition to dental implantology, these results highlight the potential of rhFGF-2 for various bone regeneration applications, including craniofacial reconstruction and fracture healing. Our study establishes rhFGF-2 as a promising stand-alone biological agent for enhancing vertical bone augmentation procedures, potentially simplifying current GBR protocols while improving clinical outcomes.

## Figures and Tables

**Table 1:** Histomorphometric analysis at 12 weeks (*n* = 7).

	Area of Newly Augmented Bone (%)		Height of Newly Augmented Bone (%)	
0.3% rhFGF-2	35.6	(9.7)	41.9	(6.7)
Control	9.1	(3.2)	13.4	(7.3)

Values are presented as mean (standard deviation). Wilcoxon signed-rank test, *p* < 0.05.rhFGF-2, recombinant human fibroblast growth factor 2.

## Data Availability

The data presented in this study are available upon request from the corresponding author.

## Abbreviations

The following abbreviations are used in this manuscript:

FGF-2	fibroblast growth factor 2
Micro-CT	micro-computed tomography
BV	bone volume
GBR	guided bone regeneration
rhFGF-2	recombinant human fibroblast growth factor 2
β-TCP	β-tricalcium phosphate

## References

[1] Di Stefano D.A., Arosio P., Capparè P., Barbon S., Gherlone E.F. (2021). Stability of Dental Implants and Thickness of Cortical Bone: Clinical Research and Future Perspectives. A Systematic Review. Materials.

[2] Tan W.L., Wong T.L., Wong M.C., Lang N.P. (2012). A systematic review of post-extractional alveolar hard and soft tissue dimensional changes in humans. Clin. Oral Implants Res..

[3] Schropp L., Wenzel A., Kostopoulos L., Karring T. (2003). Bone healing and soft tissue contour changes following single-tooth extraction: A clinical and radiographic 12-month prospective study. Int. J. Periodontics Restor. Dent..

[4] Funato A., Ishikura C., Naito K., Hasuike A. (2022). Resorbable Membrane Pouch Technique for Single-Implant Placement in the Esthetic Zone: A Preliminary Technical Case Report. Bioengineering.

[5] Lekholm U., Adell R., Lindhe J., Brånemark P.I., Eriksson B., Rockler B., Lindvall A.M., Yoneyama T. (1986). Marginal tissue reactions at osseointegrated titanium fixtures. (II) A cross-sectional retrospective study. Int. J. Oral Maxillofac. Surg..

[6] Becker W., Becker B.E. (1990). Guided tissue regeneration for implants placed into extraction sockets and for implant dehiscences: Surgical techniques and case report. Int. J. Periodontics Restor. Dent..

[7] Buser D., Urban I., Monje A., Kunrath M.F., Dahlin C. (2023). Guided bone regeneration in implant dentistry: Basic principle, progress over 35 years, and recent research activities. Periodontology 2000.

[8] Dang Y., Zhang Y., Luo G., Li D., Ma Y., Xiao Y., Xiao L., Wang X. (2024). The decisive early phase of biomaterial-induced bone regeneration. Appl. Mater. Today.

[9] Khojasteh A., Behnia H., Naghdi N., Esmaeelinejad M., Alikhassy Z., Stevens M. (2013). Effects of different growth factors and carriers on bone regeneration: A systematic review. Oral Surg. Oral Med. Oral Pathol. Oral Radiol..

[10] Che Z., Sun Q., Zhao Z., Wu Y., Xing H., Song K., Chen A., Wang B., Cai M. (2024). Growth factor-functionalized titanium implants for enhanced bone regeneration: A review. Int. J. Biol. Macromol..

[11] Farooq M., Khan A.W., Kim M.S., Choi S. (2021). The Role of Fibroblast Growth Factor (FGF) Signaling in Tissue Repair and Regeneration. Cells.

[12] Kurokawa I., Hayami J., Kita Y. (2003). A therapy-resistant chronic leg ulcer treated successfully with topical basic fibroblast growth factor. J. Int. Med. Res..

[13] Bikfalvi A., Klein S., Pintucci G., Rifkin D.B. (1997). Biological roles of fibroblast growth factor-2. Endocr. Rev..

[14] Shimabukuro Y., Terashima H., Takedachi M., Maeda K., Nakamura T., Sawada K., Kobashi M., Awata T., Oohara H., Kawahara T. (2011). Fibroblast growth factor-2 stimulates directed migration of periodontal ligament cells via PI3K/AKT signaling and CD44/hyaluronan interaction. J. Cell. Physiol..

[15] Takayama S., Murakami S., Miki Y., Ikezawa K., Tasaka S., Terashima A., Asano T., Okada H. (1997). Effects of basic fibroblast growth factor on human periodontal ligament cells. J. Periodontal Res..

[16] Kitamura M., Akamatsu M., Kawanami M., Furuichi Y., Fujii T., Mori M., Kunimatsu K., Shimauchi H., Ogata Y., Yamamoto M. (2016). Randomized Placebo-Controlled and Controlled Non-Inferiority Phase III Trials Comparing Trafermin, a Recombinant Human Fibroblast Growth Factor 2, and Enamel Matrix Derivative in Periodontal Regeneration in Intrabony Defects. J. Bone Miner. Res..

[17] Kitamura M., Nakashima K., Kowashi Y., Fujii T., Shimauchi H., Sasano T., Furuuchi T., Fukuda M., Noguchi T., Shibutani T. (2008). Periodontal tissue regeneration using fibroblast growth factor-2: Randomized controlled phase II clinical trial. PLoS ONE.

[18] Kitamura M., Akamatsu M., Machigashira M., Hara Y., Sakagami R., Hirofuji T., Hamachi T., Maeda K., Yokota M., Kido J. (2011). FGF-2 stimulates periodontal regeneration: Results of a multi-center randomized clinical trial. J. Dent. Res..

[19] Saito A., Bizenjima T., Takeuchi T., Suzuki E., Sato M., Yoshikawa K., Kitamura Y., Matsugami D., Aoki H., Kita D. (2019). Treatment of intrabony periodontal defects using rhFGF-2 in combination with deproteinized bovine bone mineral or rhFGF-2 alone: A 6-month randomized controlled trial. J. Clin. Periodontol..

[20] Fukuba S., Akizuki T., Hoshi S., Matsuura T., Shujaa Addin A., Okada M., Tabata Y., Matsui M., Tabata M.J., Sugiura-Nakazato M. (2019). Comparison between different isoelectric points of biodegradable gelatin sponges incorporating β-tricalcium phosphate and recombinant human fibroblast growth factor-2 for ridge augmentation: A preclinical study of saddle-type defects in dogs. J. Periodontal Res..

[21] Hoshi S., Akizuki T., Matsuura T., Ikawa T., Kinoshita A., Oda S., Tabata Y., Matsui M., Izumi Y. (2016). Ridge augmentation using recombinant human fibroblast growth factor-2 with biodegradable gelatin sponges incorporating β-tricalcium phosphate: A preclinical study in dogs. J. Periodontal Res..

[22] Fukuba S., Akizuki T., Matsuura T., Okada M., Nohara K., Hoshi S., Shujaa Addin A., Iwata T., Izumi Y. (2021). Effects of combined use of recombinant human fibroblast growth factor-2 and β-tricalcium phosphate on ridge preservation in dehiscence bone defects after tooth extraction: A split-mouth study in dogs. J. Periodontal Res..

[23] Kochi G., Sato S., Fukuyama T., Morita C., Honda K., Arai Y., Ito K. (2009). Analysis on the guided bone augmentation in the rat calvarium using a microfocus computerized tomography analysis. Oral Surg. Oral Med. Oral Pathol. Oral Radiol. Endodontol..

[24] Oginuma T., Sato S., Udagawa A., Saito Y., Arai Y., Ito K. (2012). Autogenous bone with or without hydroxyapatite bone substitute augmentation in rat calvarium within a plastic cap. Oral Surg. Oral Med. Oral Pathol. Oral Radiol..

[25] Kubota T., Hasuike A., Naito M., Tsunori K., Min S., Sato S. (2018). Enhancement of Bone Augmentation in Osteoporotic Conditions by the Intermittent Parathyroid Hormone: An Animal Study in the Calvarium of Ovariectomized Rat. Int. J. Oral Maxillofac. Implants.

[26] Saito Y., Sato S., Oginuma T., Saito Y., Arai Y., Ito K. (2013). Effects of nicotine on guided bone augmentation in rat calvarium. Clin. Oral Implants Res..

[27] Hosokawa R., Kikuzaki K., Kimoto T., Matsuura T., Chiba D., Wadamoto M., Sato Y., Maeda M., Sano A., Akagawa Y. (2000). Controlled local application of basic fibroblast growth factor (FGF-2) accelerates the healing of GBR. An experimental study in beagle dogs. Clin. Oral Implants Res..

[28] You S., Yu F., Fan Q., Xia T., Liang L., Yan Q., Zeng H., Shi B. (2023). Radiographic comparison of atelocollagen versus deproteinized bovine bone minerals covered with a collagen membrane in alveolar ridge preservation: A retrospective study. BMC Oral Health.

[29] Yu S.-J., Moon S.-S., Jang H.-S., Han K.-Y., Hwang K.-S., Choi S.-H., Kwon Y.-H., Kim B.-O. (2012). A clinical and histological evaluation for healing of dehiscence defects filled with an absorbable atelocollagen sponge in dogs. Tissue Eng. Regen. Med..

[30] Kobayashi N., Miyaji H., Sugaya T., Kawanami M. (2010). Bone Augmentation by Implantation of an FGF2-loaded Collagen Gel-sponge Composite Scaffold. J. Oral Tissue Eng..

[31] Kigami R., Sato S., Tsuchiya N., Yoshimakai T., Arai Y., Ito K. (2013). FGF-2 angiogenesis in bone regeneration within critical-sized bone defects in rat calvaria. Implant. Dent..

[32] Kigami R., Sato S., Tsuchiya N., Sato N., Suzuki D., Arai Y., Ito K., Ogiso B. (2014). Effect of basic fibroblast growth factor on angiogenesis and bone regeneration in non-critical-size bone defects in rat calvaria. J. Oral. Sci..

[33] Nakamura S., Ito T., Okamoto K., Mima T., Uchida K., Siddiqui Y.D., Ito M., Tai M., Okubo K., Yamashiro K. (2019). Acceleration of bone regeneration of horizontal bone defect in rats using collagen-binding basic fibroblast growth factor combined with collagen scaffolds. J. Periodontol..

[34] Bianchi S., Bernardi S., Simeone D., Torge D., Macchiarelli G., Marchetti E. (2022). Proliferation and Morphological Assessment of Human Periodontal Ligament Fibroblast towards Bovine Pericardium Membranes: An In Vitro Study. Materials.

[35] Novais A., Chatzopoulou E., Chaussain C., Gorin C. (2021). The Potential of FGF-2 in Craniofacial Bone Tissue Engineering: A Review. Cells.

